# Dynamic RFID Identification in Urban Traffic Management Systems

**DOI:** 10.3390/s20154225

**Published:** 2020-07-29

**Authors:** Bartosz Pawłowicz, Bartosz Trybus, Mateusz Salach, Piotr Jankowski-Mihułowicz

**Affiliations:** 1 Department of Electronic and Telecommunications Systems, Rzeszów University of Technology, Wincentego Pola 2, 35-959 Rzeszów, Poland; pjanko@prz.edu.pl; 2 Department of Computer and Control Engineering, Rzeszow University of Technology, 35-959 Rzeszow, Poland; 3 Department of Complex Systems, Rzeszow University of Technology, 35-959 Rzeszow, Poland; m.salach@prz.edu.pl

**Keywords:** RFID, traffic management, dynamic identification

## Abstract

The paper covers the application of Radio Frequency IDentification (RFID) technology in road traffic management with regard to vehicle identification. Various infrastructure configurations for Automated Vehicle Identification (AVI) have been presented, including configurations that can be used in urban traffic as part of the Smart City concept. In order to describe the behavior of multiple identifications of moving vehicles, an operation model of the dynamic identification using RFID is described. While it extends the definition of the correct work zone, this paper introduces the concept of dividing the zone into sections corresponding to so-called inventory rounds. The system state is described using a set of matrices in which unread, read, and lost transponders are recorded in subsequent rounds and sections. A simplified algorithm of the dynamic object identification system was also proposed. The results of the simulations and lab experiments show that the efficiency of mobile object identification is conditioned by the parameters of the communication protocol, the speed of movement, and the number of objects.

## 1. Introduction

Managing vehicle traffic in urban areas is a challenge. It concerns not only the shaping of road infrastructure in such a way that traffic flows smoothly, but also the introduction of solutions that make the route of traffic channels more flexible and make their course dependent on dynamic events occurring on the road. New tasks arising from environmental and social considerations are considered, such as servicing restricted access zones for specific vehicles. Vehicle features such as propulsion type (electric, petrol), exhaust emission standards, purpose (public transport vehicles, emergency services), as well as special access passes to private zones are considered here.

The correct identification of vehicles is therefore an important element of traffic management systems. The determination of vehicle features can be based on visual identification using surveillance cameras. However, this method has its limitations and can be unreliable in adverse weather or lighting conditions. Therefore, identification can be supported by RFID (Radio Frequency IDentification) technique using an electromagnetic field to obtain information stored in electronic form. RFID is currently used in many areas [1], such as the Internet of Things (IoT) [2], Fast Moving Consumer Goods (FMGC) in supply chains [3], and even for security and enhanced user experience at exhibitions and museums [4]. RFID, as a contactless solution, seems to fit well into tasks related to vehicle traffic. Thus, applications involving RFID, such as the maintenance of public transport [5] or the transport of goods, urban traffic management [6,7], traffic monitoring systems [8], or collecting road tolls [9,10] are currently being developed.

The use of RFID for dynamic urban traffic management is associated with the need to identify many vehicles in motion and to determine their characteristics stored in RFID transponders. New research challenges appear here, regarding the definition of the area of correct identification (interrogation zone) or the selection of appropriate communication parameters. Existing standards are constantly being updated, and new standards are being developed. This applies particularly to RFID systems using the HF and UHF ranges [9]. The identification of large groups of objects is possible due to multiple access mechanisms, resolving cases of missing or collided identifications and more.

Naturally, the identification procedure takes some finite time, which depends on the parameters of the communication protocol, such as the transmission rate from the RFID reader device to the RFID transponders and vice versa, the type of digital encoding used, etc. These factors make the identification of moving objects, such as vehicles, not obvious. Due to the limited time that transponders are present in the interrogation zone and due to the very random order of the readings, it is possible that some of them may not be identified, so some identification loss may occur. Therefore, determining how fast transponders can move and how large the interrogation zone should be is important for reliable identification.

The rest of the paper is organized as follows. Section 2 introduces general principles of RFID identification and its main elements, including the interrogation zone. Section 3 presents the use of RFID for Automatic Vehicle Identification (AVI). Various identification modes are discussed in terms of their suitability for AVI applications. In the Section 4 that follows, AVI is covered in a broader perspective known as the Smart City. The role of the traffic control system is indicated and the tasks it performs in urban traffic are discussed. The system can be supported by RFID technology, which is presented in Section 5, where various scenarios of the support are considered. Dynamic identification of many vehicles in motion requires a special approach, so the following Section 6 introduces the assumptions of the model of such a system, taking into account a dynamic interrogation zone. This section also introduces a dedicated representation of the system state, which includes sections and rounds of identification, and utilizes matrix notation. Section 7 defines the communication protocol that has been used. The protocol involves multi-access mechanisms and considers the limited inventory round time and slots with correct identification, collision, and empty ones. Section 8 presents the synthesis of the model and explains how the slot type is determined in a single round. An algorithm of the identification process is also shown. A finite and infinite stream of transponders are considered and formulas for determining of the number of lost transponders are given. The model is simulated and the results for identification efficiency are discussed in Section 7.

## 2. RFID Communication Concepts

**Figure 1:**
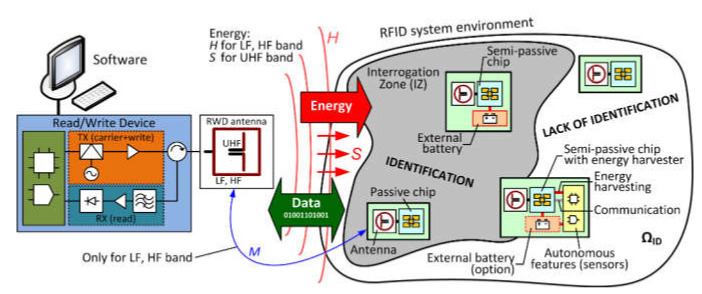
Generalized block diagram of a Radio Frequency IDentification (RFID) system.

A contactless RFID identification system involves a reader–writer device (RWD) with an antenna and one or more identified objects equipped with an RFID transponder chip (Figure 1). The transponder stores identification information and other object-related data. The RWD device reads the data from the transponder and forwards it to the supporting software. In some solutions, however, the RWD may also save or update transponder data. RWD then has a dual function that allows data transmission in two directions. In such cases, the commonly used term “RFID reader” can be misleading.

The process of radio communications in the RFID system can be carried out only in the interrogation zone (IZ, Figure 1) [11,12,13]. The presence of transponders in IZ means that after some time they will all be identified (read), as long as the system components work properly. In this situation, the identification efficiency, understood as the ratio of the number of successfully read transponders to the number of subject transponders, is equal to unity (100%). If it is assumed that there is only one electronically marked object in it, then this arrangement is called a single identification system. If there are many transponders within the interrogation zone, the communication takes place simultaneously with many transponders. In this case, an anti-collision system is used involving radio channel multi-access algorithms that allow the simultaneous differentiation of objects and arbitration in the event of data collision. In the static system, i.e., when the position of the transponder and the RWD device is fixed, the identification time is not limited (at most for justified reasons). Therefore, the definition of the interrogation zone (IZ) may be limited to a set of field and electrical conditions that must be met for a faultless identification of a group of transponders. Consequently, the interrogation zone can also be called the correct power supply zone (PSZ) for the transponders.

Typical transponders, called passive ones, contain an integrated circuit (chip) with an antenna and do not have their own power supply. Active and semi-passive transponders contain a built-in power source, such as a battery. Active transponders use the extra power to enlarge the IZ zone. Semi-passive transponders use the additional energy to perform special autonomous functions, such as measuring physical quantities (humidity, temperature, light intensity, pressure, acceleration, gas, etc. [14,15,16,17]). These additional autonomous functions do not require RWD participation to operate. It should be emphasized, however, that the additional power is not used to activate the transmission circuit, so RWD must be an active part (master) to carry out the radio communication process.

In a properly developed RFID system, all objects are successfully identified in a planned (predicted) manner. To achieve this, it is crucial to determine the maximum distance necessary to properly carry out reading/writing data from/to the transponder memory in the symmetry axis of the RWD antenna. In research studies, the synthesis of IZ often involves the analytical or experimental determination of the RFID system operation range [11]. However, in industrial practice, the “trial and error” method is usually used [18]. Moreover, this determination is adequate for single static identification. In the case of multiple identifications or a system in which identifiers dynamically change their position relative to the RWD device, as in the case of moving vehicles, determining the right system parameters is even more of a challenge.

It is worth adding that RFID identification systems usually operate in accordance with legal regulations and industrial standards. Examples are ISO14443—“Proximity integrated circuit card” [19], ISO15693—“Identification cards; Contactless integrated circuit(s) cards; Vicinity cards” [20], and ISO18000—“RFID for item management; Air interface” [21]. Part 63 of ISO18000 seems particularly suitable because it is assumed that the UHF frequency range covered by this document will also be used to identify vehicles in traffic management systems.

## 3. Smart City Traffic Solutions

Urban development is taking place along with technological progress at an ever-increasing speed. Discovering and building new solutions contributes to the evolution of not only the Smart City concept, but also the whole idea of Smart X. Initially, Smart City 1.0 focused on the sustainable technological development of cities, new technologies, stability, and control. Smart City 2.0 [22] has brought cities to a higher level by introducing a number of solutions focusing on urban development with an emphasis on environmental pollution, health, sanitation, and transport [23]. This has given residents and entrepreneurs new opportunities. However, while a small group of experts previously worked on Smart City solutions, the approach has changed in the latest generation. The evolution has gone up to level 3.0, an idea that includes a wider group of experts and is based on community–city cooperation. Urban planning is less formal, which allows the community members to get involved. While there are more and more cities that can aspire to Smart City 1.0, few can identify with the idea of Smart City 2.0 or 3.0 (e.g., Amsterdam or Rotterdam).

As the concept of the Smart City develops, new solutions are created in related areas, including Industry 4.0 or 5.0 [24]. The evolution of the Smart City 3.0 idea can be seen in a broader perspective known as Society 5.0. It brings together solutions such as Smart City, Industry, Humanity and Communication Technologies. Society 5.0 is based on certain ideas, the development of which define the functioning of individual areas. The most anticipated pillars are the evolution of connected objects, cloud computing, Big Data, autopilot vehicles, smart networks, and intelligent transportation [25]. The idea of Internet of Things, which has evolved to the Internet of Everything, is also a factor in urban development. The availability of broad communication between various types of facilities in the city thanks to LPWAN (Low Power WAN) or 5th generation telecommunications towers, access to more bands, and increased data transmission introduce new possibilities.

**Figure 2:**
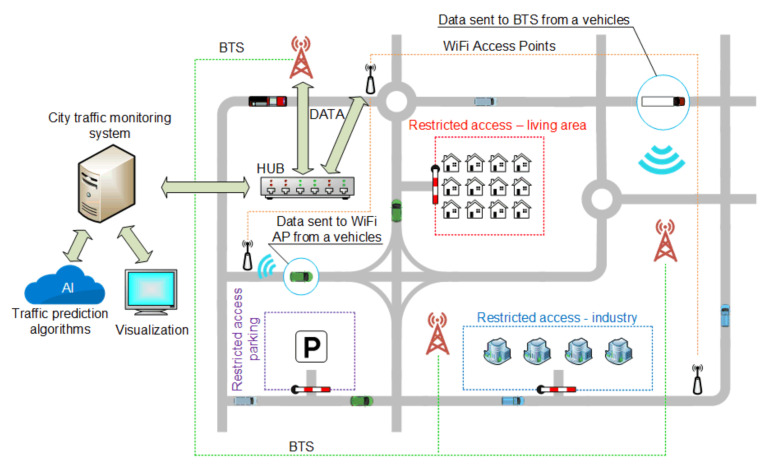
Concepts of the Smart City traffic monitoring and control system.

Transport, as one of the elements of the Smart City, is undergoing many changes to meet the assumptions of urban development and positively affect the residents [26]. Modern transport solutions include wireless communication using Wi-Fi networks, global positioning (GPS), and communication using cellular networks (Figure 2). Intelligent transport associated with the pillars of Society 5.0 also includes autonomous vehicles, traffic management, reducing the number of accidents, and ensuring a comfortable journey.

Smart Cities are built on the basis of data obtained in various ways, including sensors located in parts of the city [27], in public transport vehicles from monitoring cameras [28,29], or by using signals from cellular networks [30]. For example, data on the intensity of vehicle traffic are obtained based on indications from a mobile device (smartphone) [31], including the distance that vehicles travel in specific time intervals. According to the idea of the Internet of Things, vehicles, especially public transport vehicles, communicate with each other [32,33] and with telecommunications towers by sending data about their location. This way, a full analysis of vehicle traffic is possible, with the detection of potential accidents and a response to unusual events [34,35].

The key factor in obtaining data is the time it takes to receive it. Information sent to the system must be processed as close to real time as possible. Given the number of vehicles and devices in the city, the greatest potential for communication between the device and the management system can be dedicated closed wireless communication systems or the use of a cellular network with an emphasis on 5th generation solutions dedicated mainly to Smart Cities (Figure 2).

There are various methods for a city traffic management system to be able to retrieve the location of each vehicle in real time. The first tests used the EGRS (Electronic Route Guidance System) [36], and in the 1980s, solutions based on the Global Positioning System (GPS) appeared, which are still used today. GPS in the case of modern vehicles with built-in navigation modules works with an accuracy of about 1 m. Another solution to locate vehicles is the cellular network. Data are downloaded from devices in vehicles (built-in or portable). They send cyclical information about their location to a dedicated cloud service [37]. The location of the vehicle is determined by the location of the Base Transceiver Station (BTS) [38] usually in combination with the GPS system. Another method of determining location is a dedicated wireless Wi-Fi network with access points mounted along the road. The position relative to the access point is used to locate the vehicle.

The intelligent control of vehicle flow in urban areas is an important development direction. One of the most used solutions is the intelligent control of traffic lights. In conjunction with artificial intelligence algorithms including machine or deep learning algorithms, the supervisory system can predict the traffic load at given hours by analyzing information obtained in the previous days or weeks and performing the adequate change of traffic lights to keep traffic smooth. Intelligent transport also means parking lots (Figure 2) [39,40,41,42,43] Ticket machines operating in the concept of Internet of Things [44] provide an opportunity to analyze the parking space occupancy at certain times, and in conjunction with sensors located in parking spaces, it is also possible to determine which places are most often occupied. This allows for efficient management and appropriate modifications.

The idea of intelligent transport should include communication in both directions (two-way communication) [45]. The urban traffic management system can collect information and analyze traffic. Having information about the load on a given route, it can propose a different route for a vehicle to reach its destination faster [46,47].

Considering the rich road and highway network and increased traffic, one can understand the growing need for monitoring, controlling, and managing this traffic. This need also results from establishing separate areas with limited traffic access, which is becoming more and more common all over the world. Some examples are parts of cities that only public transport vehicles can enter or special zones only available for certain types of cars, such as hybrid or electric vehicles (EV). Such solutions require determining the type of the car, preferably automatically. This information can be crucial when entering restricted areas such as city center, underground parking, industry zone, etc. (Figure 2). Automatic Vehicle Identification (AVI) [48] is the term that describes the approach. The goal is to choose and implement a solution that will not only guarantee reliable identification of vehicles but will also allow the tracking of traffic participants in real time. In addition, it should be assumed that such solutions will also provide modern cars with credible, immediate information about the surrounding environment.

Current traffic management solutions have certain disadvantages in this respect. Monitoring based on street cameras is not fully accurate and depends on weather and lighting conditions [49,50,51,52]. Using the data from the cameras, it cannot be determined whether the vehicle is equipped with a diesel engine or whether it is electric. A visual recognition system that is used to restrict access to private or paid traffic areas may fail due to weather conditions [53]. We point out that the RFID-based traffic control system can be applied for automatic vehicle identification, as well as for improving the on-board driver assistant systems by providing environmental data.

## 4. Improving Traffic Management with RFID Solutions

The RFID technology can be used to support the management of urban infrastructure, in particular related to traffic [54]. Two options are worth considering:

- RFID transponders installed in vehicles, and RWD devices in the road infrastructure [22],
- RWD devices installed in vehicles and transponders in the road infrastructure.

In the first case, the information contained in RFID transponders in vehicles can be used for traffic management. There are two options for mounting the transponders in a vehicle: they can be placed on the windshield or integrated with the structural elements of the car during production. The factory pre-installed transponders can store information about serial numbers (e.g., VIN), spare parts and their origin, exhaust emission standards, technical inspections, and so forth. The transponders on the windshield can store access data to restricted traffic zones or parking spaces. Such data can be changed, e.g., when the owner moves to another city, changes their workplace, etc.

**Figure 3:**
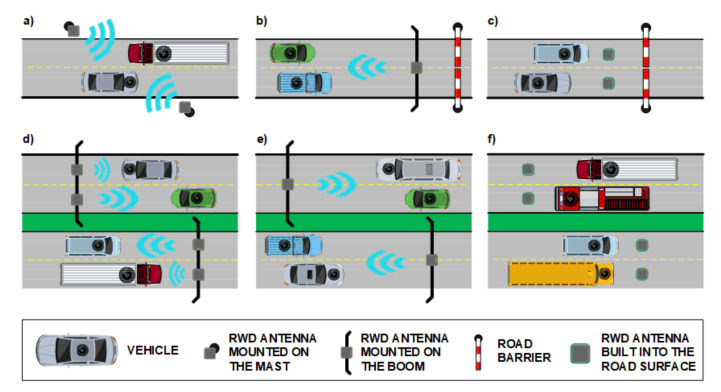
RWD antenna set-up in the traffic infrastructure: (**a**) on masts; (**b**) on boom; (**c**) built into road surface; (**d**) covering a single traffic lane; (**e**) covering multiple lanes; (**f**) built into the surface of multiple traffic lanes.

There are many options for installing hardware components in an RFID-based vehicle identification system. Figure 3 shows three possible locations of RFID antennas, depending on needs and limitations. The antennas can be placed on masts (Figure 3a) or mounted on a boom above the road (Figure 3b). They can be even built into the road surface (Figure 3c,f). This flexibility allows the use of RFID to monitor traffic and record vehicles entering or leaving restricted zones, but also to control traffic intensity. Depending on the capacity of the roadway, one or more RFID antennas should be installed. In the scenario presented in Figure 3d each antenna supports a single lane. It is also possible to reduce the number of antennas by introducing multiple AVI processes in which the antenna range covers multiple lanes (Figure 3e).

**Figure 4:**
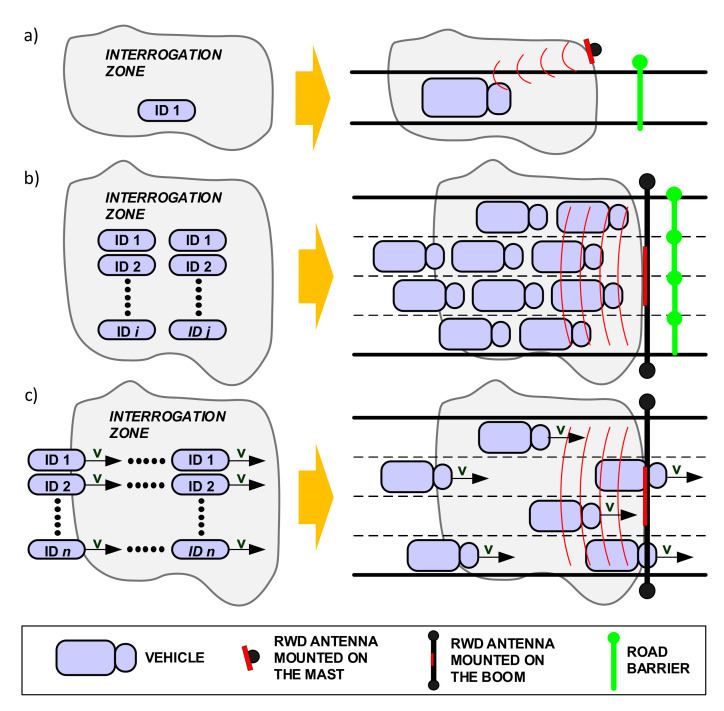
RFID identification modes: (**a**) single identification; (**b**) multiple identification; (**c**) dynamic identification.

We will now discuss the various identification modes in RFID systems and their suitability for AVI applications. In the case of single identification mode, only one vehicle present in the interrogation zone is involved (Figure 4a). This mode creates the least problems for designers, since it is not necessary to handle collision arbitration to distinguish identified vehicles. Moreover, the single identification method introduces the lowest energy demand. However, such a scenario generates problems for traffic management, e.g., how to ensure that only a particular vehicle and no other vehicles are located in the identification zone.

The solution in which multiple vehicles are identified at the same time is presented in Figure 4b. This mode is referred as anti-collision or multiple identification. Here, multiple objects (vehicles) are announce for identification. An appropriate communication protocol is required to handle the collision that occurs when the announcement comes from multiple vehicles at the same time.

The identification of moving objects introduces other challenges. This situation is shown in Figure 4c, where data is obtained from transponder s during their movement. This mode is called dynamic identification and seems to be the most desirable for AVI applications. However, dynamic identification introduces serious real-time constraints, because the time during which the identification should be carried out is limited. Obviously, the constraints come from a limited period in which the objects are staying or moving within the interrogation zone. Successful identification in this case depends on various parameters, such as speed and number of vehicles. An efficient communication protocol between RWD and transponders is also required for dynamic RFID identification. The protocol will affect the identification time in multi-access conditions. It should also recognize new objects that enter the identification zone.

**Figure 5:**
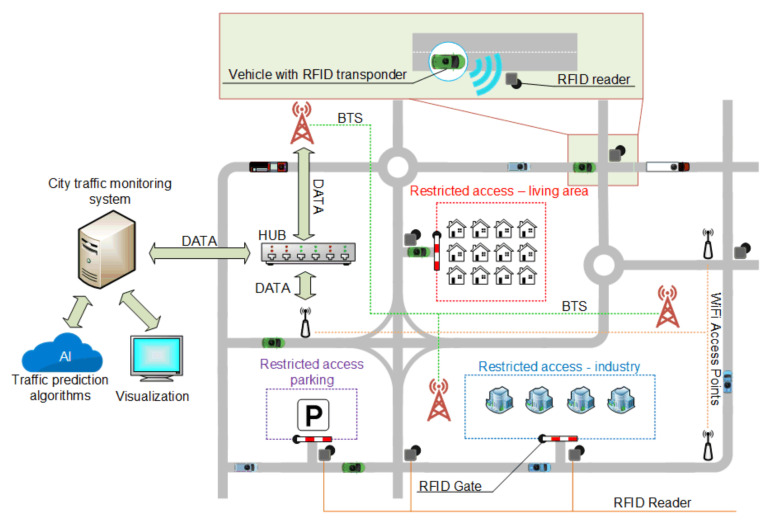
City traffic detection infrastructure with RFID transponders installed inside a vehicle.

One of the applications of RFID-based AVI can be the access control to selected zones or streets (Figure 5). In the case of electric cars, the traffic management system can let the vehicle into areas inaccessible to gasoline or diesel cars or direct it to a specially designated parking space with EV charging station. On the other hand, cars equipped with engines with LPG installations may not be allowed into zones with fire hazard or underground parking. Consider the scenario in which a vehicle is to deliver goods to an industrial recipient. The correct identification of such a vehicle before allowing its entry into the restricted zone is key to avoiding emergencies, theft, or industrial attacks. The current solutions in which transport documents or registration numbers are checked may be inaccurate. The RFID identification system can be used to create a fully automated and reliable access control system. The transponders store information that allows determining the access to specific zones. When the vehicle approaches the gate, the RFID reader detects the transponder in the vehicle and retrieves information, e.g., the identification number, name of the delivery company, vehicle type and other parameters depending on the situation. The information from the transponder is then sent to the management system and verified against the information contained in a database [55]. After verification, the system sends a reply whether the vehicle is authorized to enter the area or not. If so, gates at the entrances are opened automatically [56].

**Figure 6:**
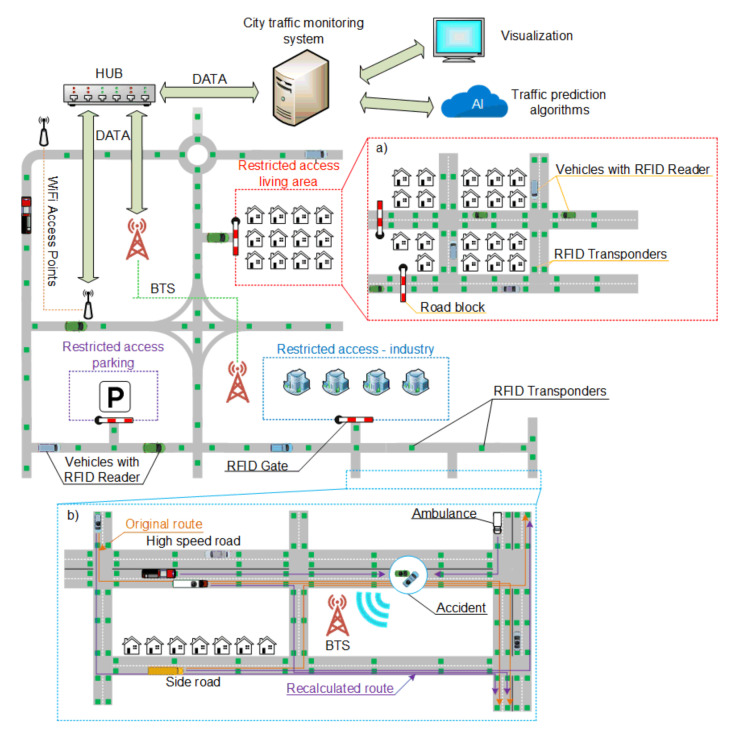
City traffic infrastructure with RFID transponders placed in the road surface: (**a**) RFID vehicle tracking, (**b**) the detection of an accident and route recalculation.

Figure 6a relates to a solution in which RFID transponders are mounted in the road infrastructure. This concept is a completely new approach in which the vehicle has a built-in RFID reader that cooperates with the communication system and the on-board computer. This way, a communication channel is created in which the vehicle receives data transmitted by the road infrastructure. RFID transponders (semi-active in this case) may provide data such as surface conditions and air or road surface temperature. Importantly, vehicle location is available even in areas where the GPS is not working.

Traffic analysis in this case may be performed using data obtained from the infrastructure. The car passes through the first RFID identifier in the road (A.1), and then after covering a certain distance the vehicle moves over to the next RFID identifier (A.2). The management system receives information about the driving time between points A.1 and A.2. Based on this data, it may determine the traffic load on a given road [8,46,57,58]. Data read from transponders placed in the road infrastructure are sent to the management system by the vehicle’s on-board computer. It is worth noting that the system can obtain information about the traffic volume in real time, because it is not based on data history, but actual RFID readings. Having real-time traffic volume data allows route correction and optimal traffic management. For example, in the case of increased intensity on a selected section of the road, the system can send information to the approaching vehicles to use detours.

One of the common traffic situations is road accidents. Even a minor incident affects the flow of traffic. Usually, a certain period of time passes before car navigation gets information about a collision and a traffic block. The use of RFID allows for immediate response as illustrated in Figure 6b. If an accident is detected, the car sends a signal to the traffic management system together with the location read from the last identifier the vehicle has passed. After obtaining information about the accident, the system will automatically notify the emergency services, which will be able to immediately go to the accident site. The system may also update the navigation routes of all vehicles whose routes have been affected by the accident. This will reduce traffic jams and increase safety as there will be no bystanders at the scene.

In the above solutions, the RFID readers and transponders move relative to each other dynamically. Therefore, for their correct operation and implementation, it is necessary to specify the system parameters in such a way that the transponder reading is smooth, and the probability of data loss is as low as possible.

## 5. The Idea of the RFID System Dynamic Model

### 5.1. Dynamic RFID Interrogation Zone

The definition of interrogation zone given in Section 2 is not sufficient for mobile object identification systems. In other words, the correct power supply zone (PSZ) does not have to be the interrogation zone (IZ). Consider an example of a finite area in which all the above conditions are met and in which, due to the high speed of moving objects equipped with transponders, only some of them are read. In the extreme case, no transponder can be read, so intuitively the interrogation zone does not exist. However, in the rest of the paper we will use the PSZ abbreviation for consistency.

Therefore, when identifying moving objects, the determination of the interrogation zone should still include the effectiveness of identification (required or obtained). The measure of this effectiveness can be the ratio of the number successfully read transponders to the number of transponders that have been identified over a given period of time. Such a measure may also be the probability of correct reading of the entire selected group of transponders. Therefore, a revised and extended definition of IZ can be proposed [59], accounting for the specifics of identifying mobile objects. The dynamic RFID interrogation zone is an area in which field, electrical, and communication conditions are met, and in which it is possible to achieve the assumed object identification efficiency.

There are a number of studies that take into account the power supply conditions for dynamic RFID systems, e.g., [12,60,61], while this work is to emphasize that communication aspects are also important and they can even change the classic definition of the interrogation zone. Given the above, an important task seems to be to create a dynamic identification system operating model useful for AVI systems. The elements of such a model created for simulation purposes are presented below.

### 5.2. Model Synthesis Assumptions

Typical RFID systems perform identification in cycles by sending RWD queries and transponder responses in the so-called inventory round. The round is a session of RWD queries and individual transponder responses according to the defined communication protocol. The rounds are repeated with the specified time cycle [62]. During their duration, the objects “enter” into the PSZ, pass through it, and leave this area. During one round, the transponders move a certain distance Δ*d*:

(1) $$\Delta d=v*T_{r}$$

where: *v* is transponder speed and *T_r_* is the duration of the inventory round.

The area equal in width to the PSZ created by moving transponders during one round is called a section in the presented approach. The sections are stationary and do not move with the transponders.

**Figure 7:**
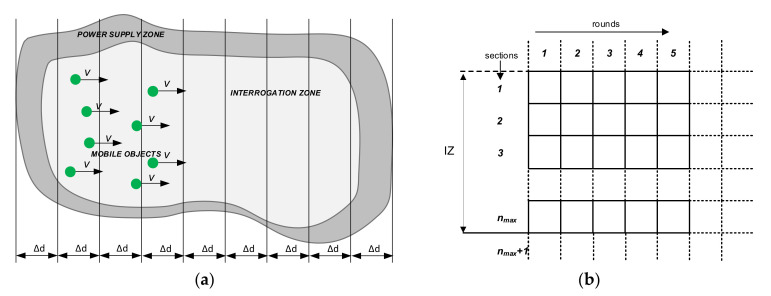
Representation of dynamic RFID system: (**a**) power supply zone (PSZ) in discrete form—divided into individual sections; (**b**) the general structure of matrices used to describe the state of the RFID system.

The dynamic RFID system power supply zone can therefore be divided into a number of sections (Figure 7a). The number of sections may generally be incomplete, which will be considered later. Sections can be sequentially numbered as 1, 2, 3, etc. The first will be the “input” section of PSZ, into which transponders are entered and where the identification process begins. Let the last section of the correct power supply area have the number *n_max_*:

(2) $$n_{max}=E\left(\frac{d_{PSZ}}{\Delta d}\right)$$

where the symbol *E*(*·*) means the integer part and *d_PSZ_* is the size of the power supply zone. Due to the cyclical operation of the system and the necessary simplifications conditioning the development of an effective model, it has been assumed that the transponder movement in the power supply zone is abrupt—the transponders enter subsequent sections, stop (and then the identification process is in progress), and after a certain duration of the round, they are transferred to the next sections, after which the whole process is repeated.

If the identified objects enter the PSZ in the number of *p_IDs_* per unit of time (per second), then their number of *p_IDr_* will enter this area during one round:

(3) $$p_{IDr}=p_{IDs}*T_{r}$$

To facilitate the presentation of further considerations, it is worth introducing the convenient concept of system state. The status of the dynamic RFID system is the number of unread transponders that are currently in the correct power supply area of this system. The system state is a function of time and may change in subsequent rounds. The state consists of the number of unread transponders in individual sections, so one can also take into account the state of individual sections in subsequent rounds.

During the dynamic identification process, the system state in the spatial and temporal domain should be analyzed. This is because during the identification process, the transponders located throughout the PSZ respond to RWD queries in a single round. Therefore, some basic properties of the system should be taken into account to facilitate the formulation of its model. They are as follows:

- Transponders in the entire PSZ are identified in a round.
- The probability of identifying a transponder does not depend on its location in the PSZ. This is because the order in which transponders are read depends on the random numbers generated at the beginning of each round.
- The number of unread transponders in a moving group decreases over time (due to correct identification), i.e., the further the transponder group enters the PSZ, the smaller the number of unread transponders in this group.
- For a given number of transponders read in a round, their number per section is proportional to the (previous) number of unread transponders in this section.

### 5.3. Representation of the System State

The system state varies between sections and rounds. Therefore, it is advisable to adopt a method of analyzing the system operation in such a way that it is possible to take into account its state both in different rounds and in different sections of the PSZ. For this reason, the approach proposed in [63] is further developed in this paper, where the analysis concerned only subsequent sections; namely, the system state representation has been added in subsequent rounds.

We will use matrix notation to represent the system state in sections and rounds, which is very convenient and creates new possibilities for analysis. It is generally assumed that the columns of the matrix will correspond to the next rounds of identification, while the rows will correspond to the next sections (Figure 7b). The dimensions of the PSZ are finite, therefore the number of matrix rows will be finite, whereas the number of rounds can be very large. This means that the horizontal matrix size can also be very large and increases with the duration of the identification process. However, thanks to this notation it is possible to record the status of all sections in a given round, to include the status of one section during individual rounds and to include changes in the number of transponders during their transition between successive sections in subsequent rounds.

The following matrices have been defined to describe the functioning of the system:

- **P** with the words *p*(*n, k*), which are equal to the number of unread transponders in the *n-th* section and the *k-th* round;
- **PISR** with the words *pisr*(*n, k*), equal to the numbers of correct identifications in the *n-th* section and the *n-th* round;
- **PIR** (single-line matrix) with the words *pir*(*k*), equal to the number of identifications in the entire PSZ-matrix in which the number of identifications in the *k-th* round is recorded;
- **PS** (single-line matrix) with the words *ps*(*k*), equal to the number of “lost” transponders in the *k-th* round. Lost transponders are transponders that were not read during the entire identification process—they left the PES and therefore will remain unread.

**Figure 8:**
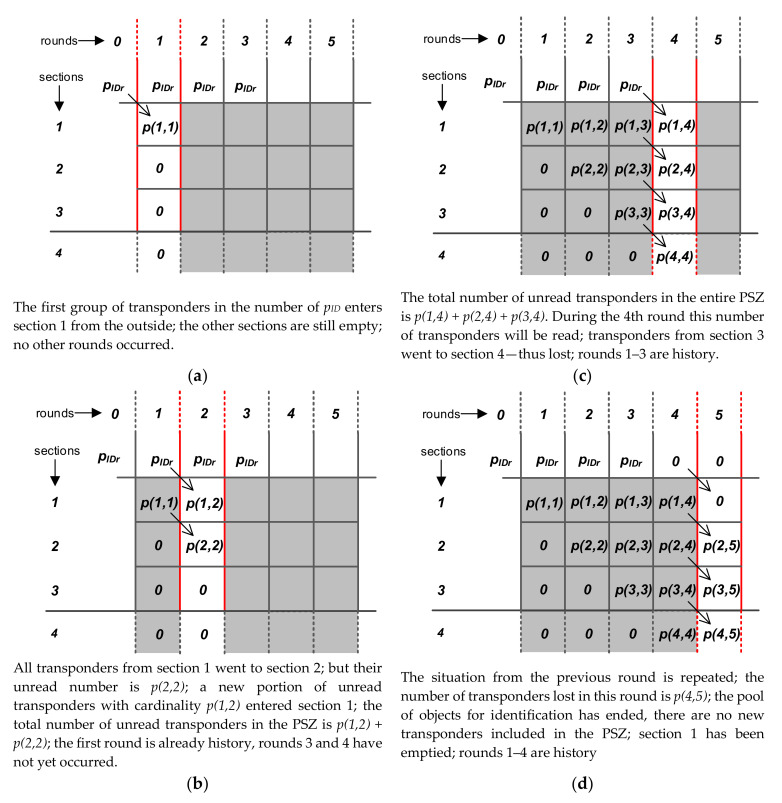
Possibilities of presenting an example of the system state in rounds and sections using matrix notation: (**a**) first round; (**b**) second round; (**c**) fourth round; (**d**) fifth round.

The task of the system is of course to read all transponders, which is why the number of lost transponders should be as low as possible. The essence of the system state representation is presented on the example of the matrix **P** in Figure 8 for the case when the PSZ contains 3 full sections, while Section 4, introduced here formally, is already outside the PSZ. The last element in the table presents the possibility of including a finite group of objects for identification. In this approach, it is relatively simple, while typical previous research assumed only infinitely long “streams” of objects for identification.

**Figure 9:**
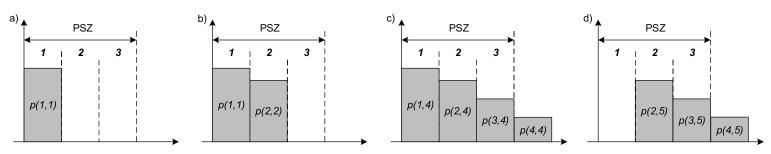
Graphic representation of the RFID system state: (**a**) first round; (**b**) second round; (**c**) fourth round; (**d**) fifth round.

It is worth noting that in addition to the matrix notation presented, the system state can be visualized in a form similar to bar graphs [63]. The system status shown in Figure 8 in four rounds can be graphically represented as in Figure 9. The areas of the marked rectangles correspond to the number of unread transponders in rounds and sections.

In representing the system state using rounds and sections, it is important to link the state of each section in a given round with the state from the previous round. This relationship can easily be described using the formula:

(4) $$p\left(n,k\right)=p\left(n-1,k-1\right)-pisr\left(n-1,k-1\right)$$

This is a record of the fact that the number of unread transponders in a given section at the beginning of a round is equal to their number in the previous section at the beginning of the previous round minus the number of identifications that occurred in the previous section and round. For formal compliance, it should be assumed that:

(5) $$p\left(0,0\right)=0;pisr\left(0,0\right)=0,$$

As follows from the model assumptions set out in p. 5.2, the total number of identifications that take place in a given round in the entire PSZ is divided into individual sections in proportions depending on the number of these sections before identification. This can be written as follows:

(6) $$pisr\left(n,k\right)=pir\left(k\right)\frac{p\left(n,k\right)}{pc\left(k\right)}$$

where *pir*(*k*) is the number of identifications in the *k-th* round in the entire PSZ, while *pc*(*k*) is the number of unread transponders in the PSZ at the beginning of this round:

(7) $$pc\left(k\right)=\overset{n_{max}}{\underset{i=1}{\sum }}p\left(i,k\right)$$

The general case of the incomplete number of sections in SZ will be considered further. The number of lost transponders in the *k-th* round can be determined from the dependence:

(8) $$ps\left(k\right)=p\left(n_{max}+1,k\right)=p\left(n_{max},k-1\right)-pisr\left(n_{max},k-1\right)$$

### 5.4. Communication Protocol in a Dynamic RFID System

To be able to read multiple transponders, the RFID system must use some kind of multi-access technique. Most modern RFID systems use techniques based on TDMA (Time Division Multiple Access) methods [64,65]. The most popular method is FSA (Frame Slotted Aloha) [64,65] and its variants. This protocol extracts frames and time slots, which partly avoids data collisions and identifies three basic events that may occur during data transmission. They include correct data transmission, collisions, and empty gaps. Data exchange between the RWD and a single transponder takes place in a single time slot. Depending on the phenomena occurring during it, several types of these gaps are distinguished. The most desirable type is the slot in which the correct data exchange session between the RWD and the transponder takes place. The multi-access method used in this paper differs from FSA in that it allows dynamic adjustment of the number of slots in the data frame. The analysis of the ALOHA system operation [66] indicates that, in the best case, the probability of identification is 38%, while the probability of an empty gap and collision is 37% and 25%, respectively. In the model of the mobile object identification system it is therefore necessary to take into account all types of gaps, because the frequency of their occurrence undoubtedly affects the time of identification of a group of objects. Depending on the number of consecutive collisions or empty gaps, the round may be shortened or lengthened to ensure the adequate number of time gaps, to avoid collision, to reduce the number of empty gaps and to increase the identification efficiency. For the algorithm demonstration, the ISO 18000-63 protocol was chosen, which is dedicated for dynamic identification.

**Figure 10:**
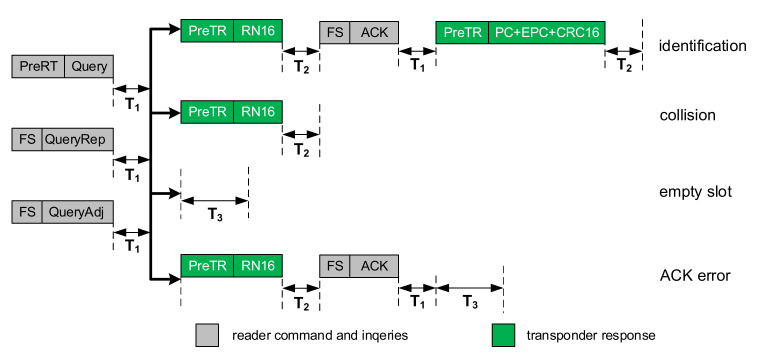
Inventory round: event types in a single round; construction of time slots in case of correct identification, data collision, empty slot, and NoACK in ISO 18000-63.

The selected ISO18000-63 protocol parameters were taken into account:

- The limited time of the inventory round. The round consists three types of slots: identification, empty and collision. Every type of slot has different duration (Figure 10). The number of all slots is limited according to *Q* algorithm. The parameter *Q* can be changed during the inventory round. In addition, the duration of each round is determined by the RWD and if the maximum round time is reached, the RWD interrupts the current round despite the actual number of slots. Otherwise, when the assumed number of slots is reached before the end of the round, the RWD waits idly until the round time elapses.
- The actual duration of every type of time slot (Figure 10). The duration of the slot depends on the time parameters of the ISO1800-63 protocol. The duration of RWD commands depends on Tari (6.25; 12.5 and 25 µs) reference time interval, the command type (*Select*, *Query*, *QueryAdjust* and *QueryRepeat*), and the type of encoding [62]. In turn, the time interval provided for transponder responses depends mainly on the type of channel coding [62], the subcarrier frequency BLF, and the type of event that occurs in the given time slot.

For simplicity, some protocol parameters are not shown here. However, they were all included in the simulation algorithm developed using all details given in [62].

## 6. Synthesis of the Model of Dynamic RFID System

### 6.1. Determination of the Time Slot Type in a Single Round

The literature often gives dependencies that determine the probability of correct identification *p_i_*, collision *p_c_* or empty slots *p_e_*, in the DFSA method [65,67] used in the ISO 18000-63 protocol. If the group of transponders *p* is read in a process in which the number of time slots in the inventory round is *L*, then the probabilities of the above-mentioned events are, respectively, [66,68]:

(9) $$p_{i}=\frac{p}{L}*{\left(1-\frac{1}{L}\right)}^{p-1},\;p_{e}={\left(1-\frac{1}{L}\right)}^{p},\;p_{c}=1-p_{i}-p_{e}$$

where *p_i_*, *p_e_* and *p_c_* are the probabilities of correct identification, empty, and collision slot, respectively. The formulas can be derived both from the basic dependencies of the probability theory and from Bernoulli’s scheme [64]. These probabilities should be understood as the probabilities of the correct reading of one transponder, the occurrence of an empty slot, or a collision in one slot of a round. The round contains *L* slots, so the expected numbers of identifications, empty slots and collisions in a round are:

(10) $$E\left(n_{i}\right)=L*p_{i}=p*{\left(1-\frac{1}{L}\right)}^{p-1}$$

(11) $$E\left(n_{e}\right)=L*p_{e}=L*{\left(1-\frac{1}{L}\right)}^{p}$$

(12) $$E\left(n_{c}\right)=L*p_{c}=L-E\left(n_{i}\right)-E\left(n_{c}\right)$$

where *E*(*n_i_*), *E*(*n_e_*), and *E*(*n_c_*) are the expected values for identification, empty slots, and collisions, respectively, in a round with length *L*. It should be remembered that these values may not be complete, whereas in a real system the number of individual slot types is complete. Therefore, in the proposed model it was assumed that for a given time slot its type will be determined in a random process with expected values equal to the values in Equations (10)–(12).

**Figure 11:**
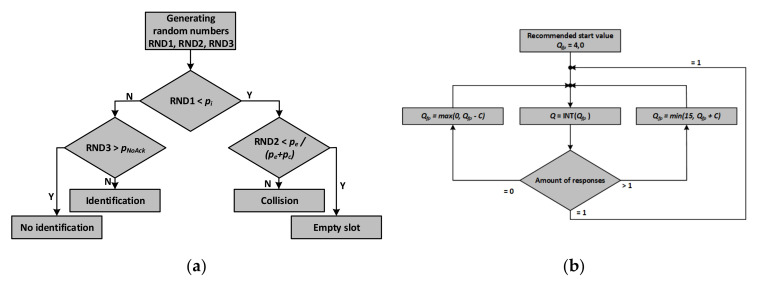
Procedures for estimating the occurrence of events in the inventory round: (**a**) algorithm of determination of events types in single slot; (**b**) The algorithm of the Q parameter determination [62].

The procedure (Figure 11a) for determining the slot type begins by generating a random number in the range (0–1). In the next step, this random number is compared with the previously determined probability of identification (apriori).

However, if identification does not occur, then there are two options left: an empty or collision slot. To select one of them, a second random number is generated and compared with the probability of these events. However, there must be conditional probabilities for the case in which no identification occurred; they are given by relationships:

(13) $$p_{e/noID}=\frac{p_{e}}{1-p_{i}}=\frac{p_{e}}{p_{e}+p_{c}}$$

(14) $$p_{c/noID}=\frac{p_{c}}{1-p_{i}}=\frac{p_{c}}{p_{e}+p_{c}}$$

determining the probabilities of an empty slot and collision provided that no identification has occurred. In addition, to make the process of selecting the slot type more realistic, a non-zero probability of a transmission error manifested as incorrect reception of the transponder’s RN16 number, which causes the transponder not responding after the ACK signal. This event is referred to as NoACK [62].

It should be noted that to estimate the number and types of slots in the inventory round, it is necessary to use a (pseudo) random number generator, because according to the regulations random number generators embedded in transponders and implementing the *Slot Counter* algorithm operate based on such distribution.

When using this method of determining the type of individual slots in the inventory round, changes in the probability *p_i_*, *p_e_*, and *p_c_* should also be taken into account during the inventory round. In many works, e.g., [63,66], in order to simplify the analysis, it is assumed that a fixed number of slots in the round is *L = 2^Q^*. In the case of the EPC Gen2 protocol, the number of time slots in the inventory round is selected by the RWD based on the Q parameter according to:

(15) $$L=2^{Q}$$

where *Q* can vary in the range of 2–15. The inventory round is initiated by the *Query* command, in which the *Q* parameter is sent to the transponders. On its basis, each transponder generates a random number from 0 to 2*^Q^* − 1 called the slot counter (*SC*), which decrements by 1 after receiving *QueryRep* commands.

Transponders that have drawn the number zero respond immediately after the *Query* command, while other transponders with *SC* number greater than zero begin arbitration and wait for subsequent *QueryRep* commands or correction of the number of slots in the *QueryAdjust* inventory round. If the EPC electronic product code is submitted correctly, the transponder goes into inventoried status. The RWD issues the *QueryRep* command starting another slot and causing the inventory flag of the previously identified transponder to change and is waiting for data from subsequent transponders.

The *QueryRep* command starts each subsequent slot in the inventory round [62]. In the event where more than one transponder replies or none of the transponders replies in a given slot, the undesirable effects discussed earlier (p. 7) may appear. Problems with two or more collisions or empty slots (none of the transponders perform data exchange) are solved by the RWD using the *QueryAdjust* commands.

The transponder that replied after its *SC* counter reached zero, but was not read correctly, decrements *SC* at the next *QueryRep* command, from 0000 h to 7FFF h. This number is so large that it prevents a later response until a new value is entered in the *SC*, and this happens at the beginning of the next inventory round or after the *QueryAdjust* command. Using this command can increase or decrease the *Q* parameter. The inventory round is then shortened or extended, and all previously unrecognized transponders participating in the inventory round generate new *SC* counter values. However, this order cannot be used to enter new transponders into the inventory round that has already begun (started with the *Select* and *Query* commands).

The *Q* parameter is selected based on the *Q_fp_*, according to the relationship *Q = INT*(*Q_fp_*). The *Q_fp_* value varies depending on the number of consecutive data collisions or empty slots. The *Q_fp_* parameter is reduced by a constant *C* if the slot is empty or increased by *C* if a data collision occurs. Depending on the value of parameter *C*, which can reach values from 0.2 to 0.5, the RWD waits for 2 to 5 empty or collided slots, then the *Q* value is incremented or decremented, and the number of slots is corrected using the *QueryAdjust* command (Figure 11b). Studies [64] indicate that the optimal value of the *C* parameter is the value calculated on the basis of the current *Q* parameter according to the relationship:

(16) $$C=\frac{0.8}{Q}$$

Changes to the *Q* parameter during a round mean that the inventory round can significantly extend over time.

### 6.2. Simulation of Single Inventory Round

Determining the slot type that currently occurs in the inventory round is the basis for building the identification model in a single round. Such a model must consider:

- dynamically determining the number of all slot types;
- parameters determining the duration of communication between RWD and transponders;
- parameter limiting the length of the inventory round and providing the ability to calculate the inventory round time.

For the above reasons, the elements of the algorithm implementing the simulation of the identification process must include additional elements responsible for the implementation of the above assumptions. The model describing a single inventory round must include, in addition to the aforementioned determination of the number of individual types of slots in the round, the duration of all types of slots.

**Figure 12:**
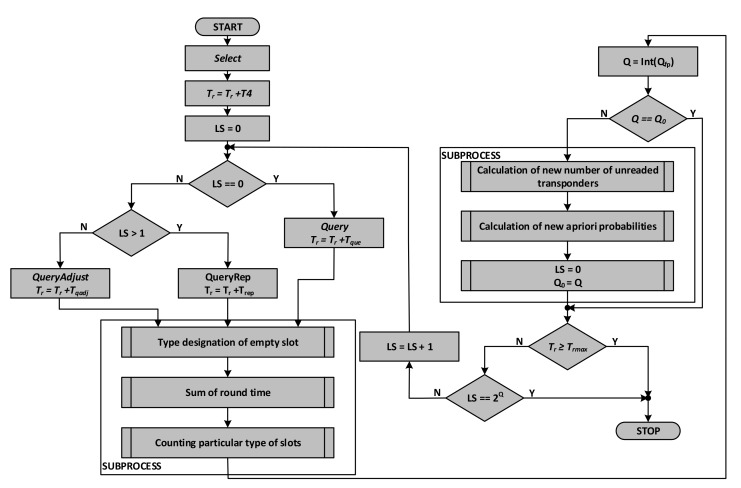
Algorithm for simulation a single inventory round.

The algorithm of a single round of the inventory simulation (Figure 12) starts with resetting the round duration time *T_r_* and including the *Select* and *Query* commands that start each round. At the beginning, the auxiliary parameter *LS* (additional time slot counter) is also reset, which is used to control the process of lengthening or shortening the round depending on the number of collisions occurring or empty slots. At this stage, the probabilities are also calculated (p. 6.1).

In the next steps they are compared with random numbers obtained using a random generator with a uniform distribution. Thanks to this, the next steps of the algorithm determine whether the correct identification has occurred or whether one of the three undesirable phenomena has occurred. Depending on which of the events took place, the duration of the specific event is added to the current duration of the round based on the data of the communication protocol parameters. In the event of a collision or empty slot, the *Q_fp_* parameter is modified to change the parameter *Q* defining the number of time slots in a given round. In the next step, the Q parameter is compared to its previous value. If this value has changed, there are a large number of collisions or empty slots in a row. If a series of empty slots has occurred, then the number of slots in the round is reduced by decrementing the value of the *Q* parameter. Otherwise, the number of slots is increased by incrementing that parameter. New probability values *p_i_*, *p_p_*, and *p_k_*. are calculated for the new value of Q and for the current number of unread transponders.

The next steps compare the round time *T_r_* with the maximum time for the round and check whether the round is to be interrupted due to exceeding this time. It is then checked whether the round has ended by comparing the current number of slots in the round with its maximum 2*^Q^* value. This number is counted since the last change of the *Q* parameter.

### 6.3. A Finite and Infinite Stream of Transponders

The RFID identification system of mobile objects can operate in conditions where a group with a finite number of objects or with an infinite stream of objects passes through the PSZ. The proposed operational model of the system includes both conditions in which new objects enter and leave the PSZ after a certain time, as well as a variant in which a specific group of transponders enters and leaves the PSZ after a certain period. Due to the fact that the stream of transponders entering the system’s operational area can have both a regular distribution (e.g., elements moving on the production line), as well as random distribution (e.g., vehicles on the highway), the main parameters defining this stream are here the speed of movement of identified objects and the rate of their entry into the area of correct operation given in transponders per second.

**Figure 13:**
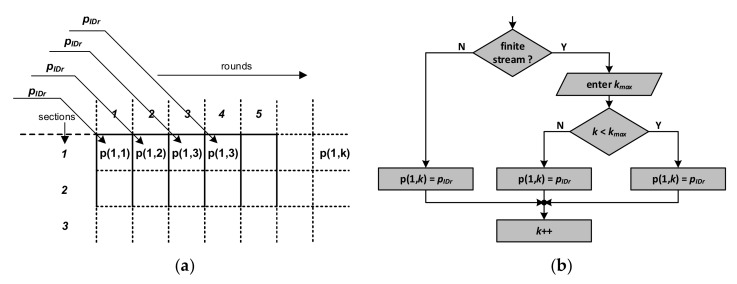
Method of handling the transponder stream: (**a**) introduction of new transponders into the first section of PSZ in subsequent rounds in the adopted matrix representation of the system state; (**b**) algorithm for simulating the infinite and finite stream of transponders entering the PSZ.

Due to the above method of representing the system state by means a set of matrices, entering both the finite and the infinite stream of transponders into the model is relatively easy to implement. Figure 13a presents the introduction of new transponders in the PSZ in the number of *p_IDr_* transponders per round. This number is always written in the subsequent columns of the first row of the **P** matrix, which corresponds to Section 1 of the area. The transponder stream simulation can therefore be presented as a fragment of the algorithm shown in Figure 13b.

In the first step, the number of *k_max_* slots is determined. This parameter determines the number of rounds in which the first transponders appear in Section 1. The finite stream condition is then checked. If the stream is infinite (*k_max_* has not been determined), then the next **P**-matrix cells, i.e., *p*(*1, k*), will enter the *p_IDr_* value of transponders that entered the first section of the PSZ. The value of *k* is then incremented, and the next batch of transponders is assigned to Section 1 in the next round. If the *k_max_* parameter takes a finite value, after reaching and then exceeding the *k_max_* value, the first number of transponders enter the first sections of subsequent rounds.

### 6.4. Determination of the Number of Lost Transponders in a Round

In the simulation of the operation of the mobile object identification system, it is necessary to determine whether all transponders located in PSZ will be read. In other words, the algorithm for simulating such a system should implement a method for estimating transponder losses in a round, taking into account the specifics of the PSZ discretization method used, so that the border of this area does not always coincide with the border of the last section.

**Figure 14:**
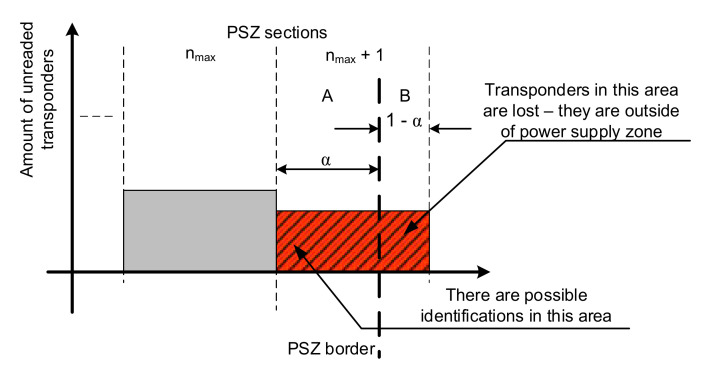
Illustration of the idea of estimating the number lost transponders.

To describe this phenomenon, it is necessary to approximate the phenomena occurring at the PSZ border. Figure 14 shows the situation in which the last section marked *n_max_* + 1 is only partially in the PSZ. Part “A” of this section is in the PSZ, while part “B” is already outside it. To correctly estimate the number of objects lost at the boundary of the area, an α coefficient has been introduced to determine which part of the last section is in the PSZ.

This ratio is therefore the ratio of the size of the ‘A’ part of the last section to the size of the entire section; it can be described by:

(17) $$\alpha =\frac{d_{PSZ}-n_{max}*\Delta d}{\Delta d}$$

The need to introduce this coefficient can be explained by the fact that despite the adopted representation and recording the system state at the beginning of a given round, identifications may still occur in the ‘A’ part of the section *n_max_* + 1 during the round. Thus, the loss of transponders in the entire round is equal to the sum of the number of transponders in part “B” of the section *n_max_* + 1 and the number of transponders not read from part “A” of this section. As such, if the total number of transponders in the PSZ is:

(18) $$pc\left(k\right)=\overset{n_{max}}{\underset{i=1}{\sum }}p\left(i,k\right)+\alpha *p\left(n_{max}+1,k\right)$$

and the total number of identifications in a given inventory round *k* is *pir*(*k*), the number of lost transponders *ps*(*k*) can be written using the formula:

(19) $$ps\left(k\right)=p\left(n_{max}+1,k\right)*\left(1-\alpha \right)+p\left(n_{max}+1,k\right)*\alpha -pir\left(k\right)\frac{p\left(n_{max}+1,k\right)*\alpha }{pc\left(k\right)}$$

which means that the number of transponders lost in round *k* is equal to the sum of transponders not read in parts ‘A’ and ‘B’ of the section *n_max_* + 1 minus the number of transponders read during this round in part ‘A’. After the necessary transformation, this relationship can be written as:

(20) $$ps\left(k\right)=p\left(n_{max}+1,k\right)*\left[1-\alpha \frac{pir\left(k\right)}{pc\left(k\right)}\right]$$

**Figure 15:**
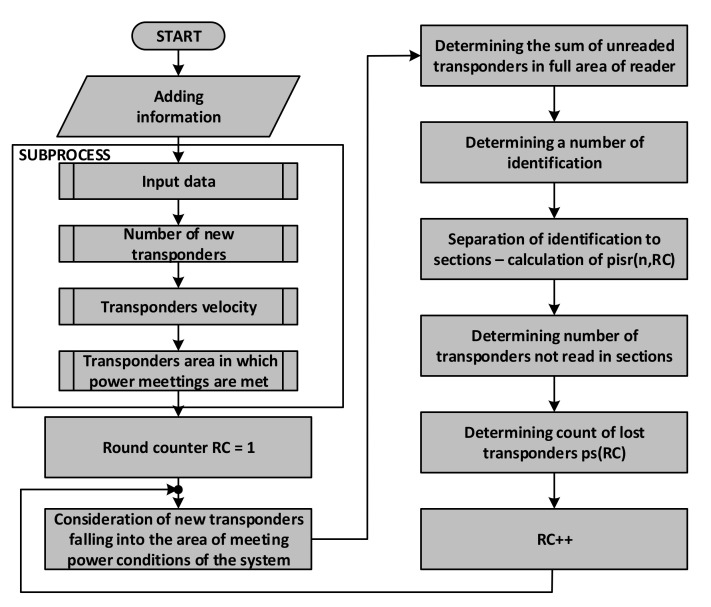
A simplified diagram of the identification system procedure using the proposed model.

On the basis of the presented dependencies, one can create a block diagram of the algorithm for simulation of dynamic RFID system operation (Figure 15).

## 7. Identification Efficiency Tests and Results

Based on the simulations carried out, the relationship between the number of transponders entering the area of correct operation per unit of time and the size of PSZ at a given probability has been determined, and between the speed of transponder groups and the size of this area. The correct working area was established for the probability of identification assumed at 99.9% and 99.99%.

**Figure 16:**
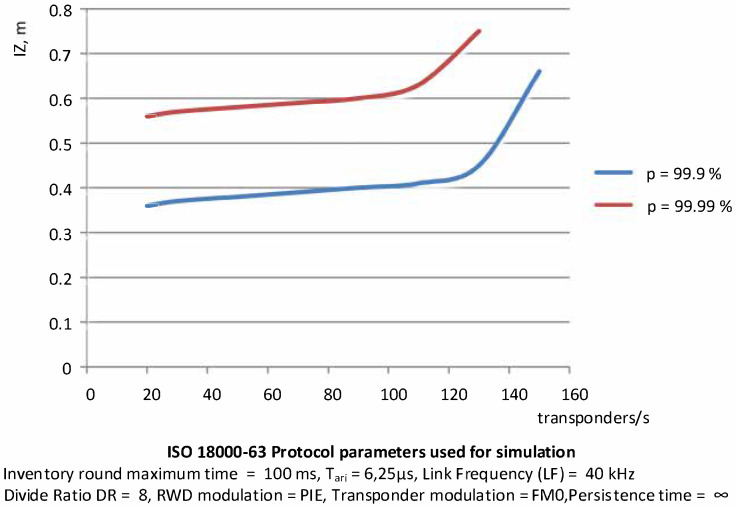
Determination of the interrogation zone depending on the number of transponders at assumed probability levels.

The characteristics presented in Figure 16 show a non-linear relationship between the size of the area and the number of transponders. It can be seen that a change in the transponder stream in the range from 20 units per second to 110 units per second does not cause a significant change to the size of the correct work area. This can be explained by the fact that the number of sections in such an area is sufficient to ensure the probability of identification at a given level. However, if the number of transponders increases above 110 per sec for *p* = 99.9%, it is necessary to increase the size of this area. This is due to the increase in the number of sections, and thus the number of inventory rounds, in order to maintain the probability of identification at a given level for more objects falling into the area.

A similar situation occurs when the probability of identification is 99.99%. In this case, the size of PSZ must be correspondingly larger. Up to 150 transponders per second falling into the PSZ can be identified with the assumed protocol parameters, or even more in the future.

**Figure 17:**
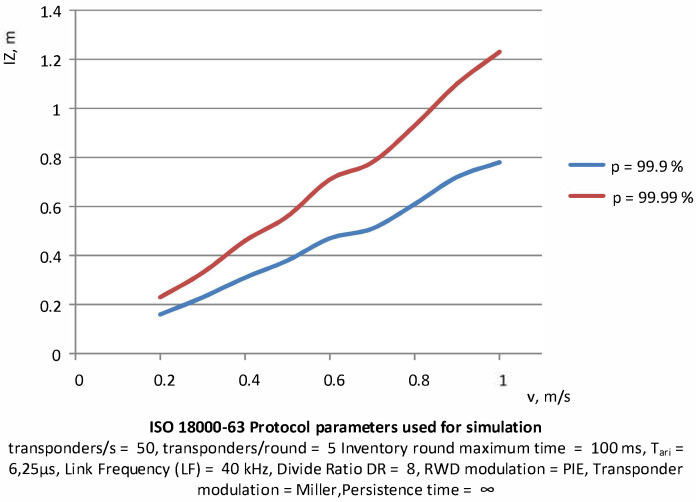
Determination of the interrogation zone in dependence on the speed of the group of transponders at assumed probability levels.

The situation is different when the size of the PSZ depends on the speed of the objects. The relationship observed in this case is similar to the linear one (Figure 17) but increasing the size of the area is necessary to obtain the probability of identification at 99.9–99.99%. As can be seen, with a slightly higher probability, the area of PSZ is significantly increased.

**Figure 18:**
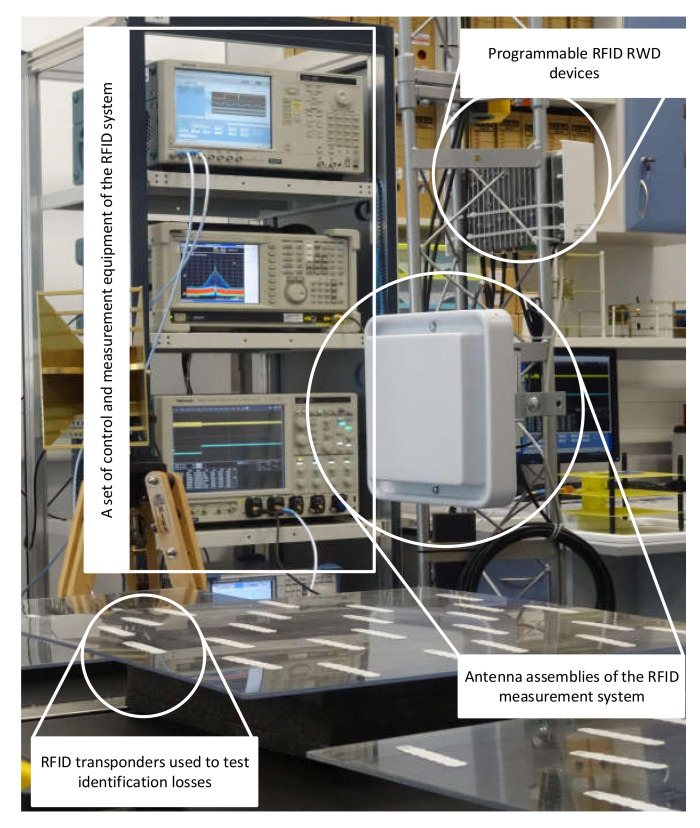
Laboratory tests of dynamic identification with multiple RFID transponders.

**Figure 19:**
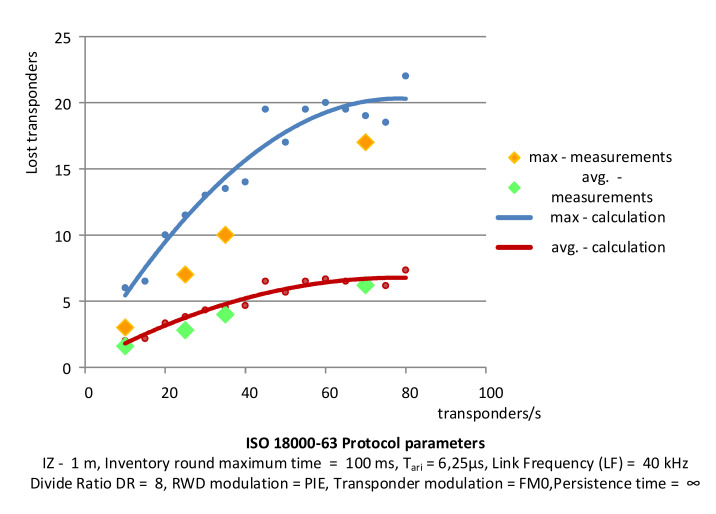
Comparison of experimental measurements of identification losses with calculations.

The experimental results were obtained on the basis of tests using a laboratory stand reflecting the flow of identifiers and selected traffic situations. Figure 18 shows the setup for testing the dynamic identification of RFID transponders. The multiple transponders are attached to the platform moving at a specific speed to test the accuracy of identification. The experimental results of identification losses are shown in Figure 19, together with calculated values. The red line indicates the approximate average value of identification losses increasing with the number of transponders entering the PSZ area obtained as a result of the calculations. The blue line represents the maximum loss of identification. The green points mark the average loss of objects obtained as a result of repeating the experiment five times, while the orange dots indicate the maximum loss of transponders obtained in the experiment. The measurements were made for groups of 10, 20, 35, and 70 transponders per second falling into the area of the correct power supply conditions.

**Figure 20:**
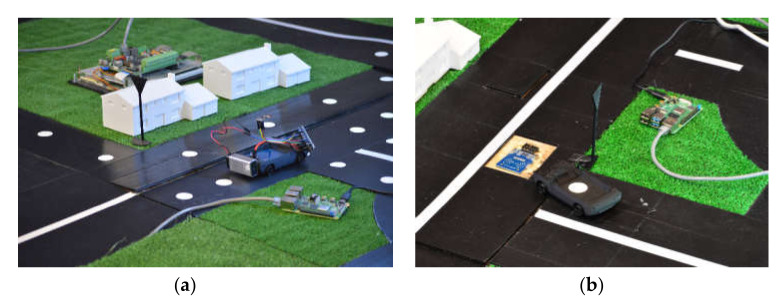
Lab traffic model with RFID infrastructure: (**a**) a vehicle equipped with RWD crossing an intersection and turning right; (**b**) a vehicle with RFID transponder passing over RWD.

Other laboratory elements allow for the testing of selected RFID application scenarios in urban traffic. Some of them are shown in Figure 20. The laboratory model of a road infrastructure measuring 3 × 3 m has two sections. In the first one, RFID transponders are placed on the road infrastructure, and the RWD devices are mounted in vehicles (Figure 20a). The second section has RWD devices installed as part of the road infrastructure, while the vehicles are equipped with transponders (Figure 20b). The experiments were performed using the Arduino Nano modules as the vehicle’s on-board computer and the Raspberry PI 4 as a hub transmitting data to the city traffic control center. Communication between RPI 4 and Arduino Nano is done via WiFi using the ESP8266 module. Data from the vehicles and RFID devices installed in the road infrastructure is sent to the cloud based Azure IoT Hub for storing and further processing.

## 8. Conclusions

Automatic identification processes in road traffic and transport increasingly require the use of modern radio identification methods. It is favored by the greater availability of electronic identifiers, namely RFID transponders, the continuous reduction of their production costs and the standardization of the operating conditions of radio identification system components. This work reviews and assesses the conditions affecting the identification process of moving objects, which are vehicles in urban traffic.

The simultaneous identification of multiple moving objects is a special challenge. A synthesis and simulation implementation of the system operation model was presented, taking into account the relationship between the number of identifiers, their movement parameters, and the size of the correct identification area, with given communication protocol parameters and identification efficiency. The result of determining the effectiveness of the dynamic identification system is the ability to determine the size of its correct work area for a given level of identification probability. In such a designated area, under given conditions, it is possible to carry out the identification process by estimating the levels of average and maximum losses.

In this work, the known definition of the correct work area has been extended. In static systems, the area of correct operation is in fact the area in which the power supply conditions for transponders are met. In dynamically operating systems, the correct operation area is, however, the resultant of the correct power supply area and the area in which the assumed identification efficiency is ensured. The article introduces the concept of dividing the PSZ for an identification of moving objects (vehicles) into sections corresponding to the cycles in which the system works (the so-called inventory rounds), and in which the objects entering the area and already present in it are identified. In order to properly estimate the number of transponders identified (read) and not identified in subsequent rounds and sections, a set of assumptions was introduced based on the basic features of the dynamic system.

The concept of system state was also introduced, and a method of its description was proposed using a set of matrices in which the numbers of unread, read, and lost transponders are recorded in subsequent rounds and sections. A simplified algorithm of the dynamic object identification system was also proposed. The results of the simulations carried out showed that the efficiency of moving object identification is conditioned by the parameters of the communication protocol, the speed of movement, and the number of objects. The simulation results were verified against laboratory experiments.

Compared to previous studies, the presented model of dynamic RFID system operation uses a number of new algorithmic and descriptive solutions:

- A convenient concept of system state was introduced.
- A computationally effective representation of the state using a matrix set was presented.
- A determination of the number of individual events in round time slots based on random processes with assumed expected values was adopted.
- It became possible to change these values during the round as the identification progressed.
- The ability to set the probability of identification failure due to transmission errors not related to collisions was introduced.
